# Role of the first WHO mutation catalogue in the diagnosis of antibiotic resistance in *Mycobacterium tuberculosis* in the Valencia Region, Spain: a retrospective genomic analysis

**DOI:** 10.1016/S2666-5247(23)00252-5

**Published:** 2024-01

**Authors:** Ana María García-Marín, Irving Cancino-Muñoz, Manuela Torres-Puente, Luis M Villamayor, Rafael Borrás, María Borrás-Máñez, Montserrat Bosque, Juan J Camarena, Ester Colomer-Roig, Javier Colomina, Isabel Escribano, Oscar Esparcia-Rodríguez, Ana Gil-Brusola, Concepción Gimeno, Adelina Gimeno-Gascón, Bárbara Gomila-Sard, Damiana González-Granda, Nieves Gonzalo-Jiménez, María Remedio Guna-Serrano, José Luis López-Hontangas, Coral Martín-González, Rosario Moreno-Muñoz, David Navarro, María Navarro, Nieves Orta, Elvira Pérez, Josep Prat, Juan Carlos Rodríguez, María Montserrat Ruiz-García, Hermelinda Vanaclocha, Valencia Region Tuberculosis Working Group, Fernando González-Candelas, Victoria Furió, Iñaki Comas

**Affiliations:** a Tuberculosis Genomics Unit, Instituto de Biomedicina de Valencia, Valencia, Spain; b Joint Research Unit Infección y Salud Pública, FISABIO-University of Valencia, Institute for Integrative Systems Biology, Valencia, Spain; c FISABIO Public Health, Valencia, Spain; d Microbiology Service, Hospital Clínico Universitario de Valencia, Valencia, Spain; e Microbiology and Parasitology Service, Hospital Universitario de La Ribera, Alzira, Spain; f Microbiology Service, Hospital Arnau de Vilanova, Valencia, Spain; g Microbiology Service, Hospital Universitario Dr Peset, Valencia, Spain; h Microbiology Laboratory, Hospital Virgen de los Lirios, Alcoy, Spain; i Microbiology Service, Hospital de Denia, Denia, Spain; j Microbiology Service, Hospital Universitari i Politècnic La Fe, Valencia, Spain; k Microbiology Service, Hospital General Universitario de Valencia, Valencia, Spain; l Microbiology Service, Hospital General Universitario de Alicante, Alicante, Spain; m Microbiology Service, Hospital General Universitario de Castellón, Castellón, Spain; n Microbiology Service, Hospital Lluís Alcanyis, Xativa, Spain; o Microbiology Service, Hospital General Universitario de Elche, Elche, Spain; p Microbiology Service, Hospital Universitario de San Juan de Alicante, Alicante, Spain; q Microbiology Service, Hospital de la Vega Baixa, Orihuela, Spain; r Microbiology Service, Hospital Francesc de Borja, Gandía, Spain; s Subdirección General de Epidemiología y Vigilancia de la Salud y Sanidad Ambiental de Valencia, Valencia, Spain; t Microbiology Service, Hospital de Sagunto, Sagunto, Spain; u CIBER of Epidemiology and Public Health, Madrid, Spain

## Abstract

**Background:**

In June, 2021, WHO published the most complete catalogue to date of resistance-conferring mutations in *Mycobacterium tuberculosis*. Here, we aimed to assess the performance of genome-based antimicrobial resistance prediction using the catalogue and its potential for improving diagnostics in a real low-burden setting.

**Methods:**

In this retrospective population-based genomic study *M tuberculosis* isolates were collected from 25 clinical laboratories in the low-burden setting of the Valencia Region, Spain. Culture-positive tuberculosis cases reported by regional public health authorities between Jan 1, 2014, and Dec 31, 2016, were included. The drug resistance profiles of these isolates were predicted by the genomic identification, via whole-genome sequencing (WGS), of the high-confidence resistance-causing variants included in the catalogue and compared with the phenotype. We determined the minimum inhibitory concentration (MIC) of the isolates with discordant resistance profiles using the resazurin microtitre assay.

**Findings:**

WGS was performed on 785 *M tuberculosis* complex culture-positive isolates, and the WGS resistance prediction sensitivities were: 85·4% (95% CI 70·8–94·4) for isoniazid, 73·3% (44·9–92·2) for rifampicin, 50·0% (21·1–78·9) for ethambutol, and 57·1% (34·0–78·2) for pyrazinamide; all specificities were more than 99·6%. Sensitivity values were lower than previously reported, but the overall pan-susceptibility accuracy was 96·4%. Genotypic analysis revealed that four phenotypically susceptible isolates carried mutations (*rpoB* Leu430Pro and *rpoB* Ile491Phe for rifampicin and *fabG1* Leu203Leu for isoniazid) known to give borderline resistance in standard phenotypic tests. Additionally, we identified three putative resistance-associated mutations (*inhA* Ser94Ala, *katG* Leu48Pro, and *katG* Gly273Arg for isoniazid) in samples with substantially higher MICs than those of susceptible isolates. Combining both genomic and phenotypic data, in accordance with the WHO diagnostic guidelines, we could detect two new multidrug-resistant cases. Additionally, we detected 11 (1·6%) of 706 isolates to be monoresistant to fluoroquinolone, which had been previously undetected.

**Interpretation:**

We showed that the WHO catalogue enables the detection of resistant cases missed in phenotypic testing in a low-burden region, thus allowing for better patient-tailored treatment. We also identified mutations not included in the catalogue, relevant at the local level. Evidence from this study, together with future updates of the catalogue, will probably lead in the future to the partial replacement of culture testing with WGS-based drug susceptibility testing in our setting.

**Funding:**

European Research Council and the Spanish Ministerio de Ciencia.

## Introduction

With approximately 750 000 global cases every year, drug-resistant tuberculosis has devastating effects not only on population health but also on the budgets of public health systems.^1^, ^2^ Personalised treatment of patients with tuberculosis, a keystone in the control of the disease, is becoming a reality thanks to a combination of advances in genomic-based drug susceptibility tests and the extended use of molecular diagnostics at the point of care. In the past 10 years, studies linking mutations in the *Mycobacterium tuberculosis* genome to phenotypic resistance have proliferated with an exponentially growing number of strain genomes and associated phenotypes available.^3^, ^4^ In June, 2021, WHO released the first catalogue of mutations associated with phenotypic resistance based on culture drug susceptibility results from 38 000 isolates worldwide.^5^, ^6^ The catalogue identifies mutations associated with resistance to all first-line drugs (ie, isoniazid, rifampicin, ethambutol, and pyrazinamide) and some second-line antibiotics, but also variants not associated with resistance, including interim calls obtained using reliable testing methods not yet endorsed by WHO, for rare mutations. This catalogue is the most complete and detailed reference list to date and can aid in boosting the development of genome-based resistance diagnosis worldwide.

**Research in context**

**Evidence before this study**

In the past 8 years, the potential of whole-genome sequencing (WGS) to predict resistance has been essential in the development of new tools for drug-resistant tuberculosis diagnostics. Our main source of previous scientific evidence was the PubMed database. We searched for publications in all languages from database inception until July 30, 2022, and we used a combination of the following terms: “whole genome sequencing”, “*Mycobacterium tuberculosis”*, “genome-based resistance prediction”, “drug-resistant tuberculosis”, and “phenotypic drug susceptibility testing”. Four studies had evaluated the performance of genome-based resistance prediction in *Mycobacterium tuberculosis*. Two of these studies included clinical isolates from a single low-burden country, the Netherlands (n=1136), and from a high-burden setting, southern India (n=223). The other two studies were the CRyPTIC Consortium multicentre study, with 23 collections of *M tuberculosis* (n=10 290) from 16 different countries, and a software tool evaluation, which included some of the CRyPTIC collections plus two other independent sets (n=10 207) that were enriched for antimicrobial resistance. All the studies except from the study from southern India had reached high values of sensitivity and specificity for the four first-line antituberculosis drugs. However, none of the studies had applied the WHO catalogue of resistance-associated mutations in *M tuberculosis*, which is currently the most comprehensive list of mutations based on phenotypic data. Apart from the initial assessment of the WHO catalogue with the training set (n=38 215), only one additional study had specifically assessed the performance of the catalogue using a curated dataset (n=8321) with *M tuberculosis* sequences from more than ten high-burden drug-resistant tuberculosis countries. The additional study had sensitivity and specificity values lower than the ones reported by the WHO-associated publication.

**Added value of this study**

Since the WHO catalogue of resistance-conferring mutations in *M tuberculosis* was released, it has still not been tested in a region-specific clinical setting. To our knowledge, our study provides the first insight into the performance of the catalogue in a real-world scenario. This insight is essential to understand how to enhance the catalogue for reaching a full integration of WGS resistance prediction in drug-resistant tuberculosis diagnostics. Our results reveal that the catalogue enables a highly accurate detection of resistance to the first-line drugs, even though some resistant cases are still missed. Furthermore, we have shown that the clinical use of this genomic data has a positive effect on the diagnosis of drug-resistant tuberculosis, even in a low-burden setting.

**Implications of all the available evidence**

The caveats of culture-based drug susceptibility testing (DST) suggest that there is an urgency to complement phenotypic assays with genomic data to be able to move towards pathogen-based personalised treatments. WHO currently supports the use of a composite standard in resistance diagnostics, which involves the use of expert guidelines to determine whether the phenotypic DST or the WGS result should prevail depending on the antibiotic and the specific mutation. Our results highlight that the WHO catalogue is a reliable reference standard for genome-based resistance prediction that can boost the implementation of WGS in drug-resistant tuberculosis diagnostics. However, the catalogue should be constantly updated by WHO and expanded with new evidence to meet the WHO desired target product profile. For this reason, we recommend the routine use of WGS in clinical samples, in parallel with culture-based DST, which would help identify new putative resistance-conferring mutations and increase our knowledge of the association between phenotypic resistance and mutations in *M tuberculosis*.

In parallel to genomic-based methods, drug susceptibility testing (DST) for diagnostics has also evolved. Phenotypic DST (pDST) has long been the gold standard for DST. However, pDST poses three main technical problems: (1) it is unreliable and reproducibility is poor for some antibiotics such as pyrazinamide and ethambutol,^7^ (2) some resistance-conferring variants, particularly the disputed *rpoB* mutations, sometimes yield a negative pDST result,^8^ and (3) the antibiotic concentrations tested are not a perfect predictor of clinical outcome.^8^, ^9^, ^10^ As a consequence, in the past 2 years, WHO has recommended changes in the critical concentrations of drugs such as rifampicin to accommodate some of these limitations.^8^ Moreover, pDST requires long periods of incubation, high-level biosafety facilities, and technical expertise. To expand availability and reduce the time of drug-resistant tuberculosis diagnosis, WHO endorsed the use of rapid nucleic acid amplification tests, which are easy to use and less time-consuming. However, these tests are limited to the detection of a few common pre-selected mutations conferring resistance and for a specific set of antitubercular drugs.^11^

Current guidelines of drug-resistant tuberculosis diagnosis are progressing towards composite reference standards that combine both phenotypic and genotypic data.^12^ Although in some health systems genome-based resistance prediction has been successfully applied in the routine diagnostic workflow of patients with tuberculosis, its use is mostly limited to complementary diagnostics.^13^, ^14^ This limitation is mainly due to an absence of global standardisation of bioinformatic analyses and data interpretation and access to the technology, particularly in low-income and middle-income countries.^15^, ^16^ In this context, the WHO mutation catalogue establishes a starting point for a harmonised development of whole-genome sequencing (WGS) resistance prediction. Here, we aimed to assess the performance of the new catalogue on genome-based resistance prediction in a real low-burden setting. For this aim, we analysed the clinical samples belonging to a population-based dataset from the Valencia Region, Spain, reported between 2014 and 2016 for which first-line pDST data at that time were also available. The region has around 5 million inhabitants including a 14% non-Spanish-born population. Some of the countries among the top ten major contributors to foreign nationals are also high-burden or mid-burden settings of tuberculosis. An additional aim of this study was to use knowledge available in 2022 to understand the potential of genomic prediction for enhancing the management of patients with drug-resistant tuberculosis even in a low-burden region.

## Methods

### Sample collection and study design

In this retrospective population-based genomic study *M tuberculosis* isolates from 25 clinical laboratories in the Valencia Region, Spain, were collected. The study included all culture-positive tuberculosis cases reported from Jan 1, 2014, to Dec 31, 2016, by the regional public health authorities. No other inclusion or exclusion criteria were applied to the isolates analysed in this study. Only a single sample (the earliest positive mycobacteria growth indicator tube or Löwenstein–Jensen culture) from each patient was included. The pDST was performed by the peripheral biosafety level 3 laboratories for routine patient-care purposes using standard procedures (appendix 1 p 1).

The study obtained the approval of the Ethics Committee for Clinical Research from the Valencia Regional Public Health Agency. Informed consent was waived because tuberculosis diagnosis is part of the regional mandatory surveillance programme of communicable diseases. All personal, epidemiological, and clinical information from patients was anonymised, and patient identification data has not been maintained.

### DST using the resazurin microtitre assay

We used the resazurin microtitre assay to reassess the drug susceptibility for isoniazid and rifampicin of a series of samples of interest.^17^ We also included H37Rv in the assay as the control reference strain of *M tuberculosis*. Briefly, we grew the samples in 7H9 broth (Becton Dickinson, Franklin Lakes, NJ, USA) with OADC growth supplement (Becton Dickinson) and 0·05% Tween-80 (Becton Dickinson Difco, Franklin Lakes, NJ, USA) to an optical density of 0·5. We prepared a 96-well plate with serial dilutions of each antibiotic (0·016–1·000 mg/L for rifampicin and 0·06–4·00 mg/L for isoniazid, both in two-fold steps) in 7H9 supplemented with OADC. We inoculated 10^4^ bacteria per well for both antibiotics and two replicates per plate. We added 20 μl of 0·02% resazurin (Acros Organics Thermo Scientific Chemicals, Waltham, MA, USA) to each replicate after 7 and 14 days of incubation at 37°C, and incubated for a further 24 h. Plates were inspected visually after 24 h and the colour change of the resazurin was recorded, determining that the minimum inhibitory concentration (MIC) was the lowest one where the resazurin remained blue.

### WGS and drug susceptibility prediction

DNA from positive tuberculosis diagnostic cultures was extracted using a standard CTAB-based protocol.^18^ Sequencing libraries were constructed with Nextera XT DNA Library Prep Kit (Illumina, San Diego, CA, USA) and sequenced on the Illumina MiSeq platform (Illumina), using standard procedures. Generated paired-end sequencing reads were filtered using Kraken software (version 0.10.5)^19^ to keep only those reads belonging to the *M tuberculosis* complex.

The bioinformatic analysis (mapping and variant calling) was performed following a previously described and validated pipeline comparable to that of major national public health tuberculosis reference laboratories^20^ using specific parameters.^21^ The genotype prediction was performed comparing our single-nucleotide polymorphism and indel data with the WHO catalogue of drug-resistant-associated mutations in the *M tuberculosis* complex.^5^ The resistant or susceptible status of the isolates for each antibiotic was predicted according to the presence or absence of resistance-conferring mutations and their grade of association with resistance reported in the catalogue (appendix 1 pp 1–2).

Phenotypically resistant isolates with no known resistance-conferring mutations were screened manually to identify new candidate resistance-associated mutations, including indels (appendix 1 p 2). The mutation list obtained by scanning a list of secondary genes that are likely to contain resistance mutations was filtered to remove positions indicative of phylogenetic markers in two ways: (1) we made sure the mutation was not present in other susceptible samples in the dataset under study, and (2) we searched for the variant in the WHO catalogue, which provides information for more than 17 000 mutations. We systematically reviewed previous literature on the possible association with resistance of the remaining variants. Finally, we proposed a list of novel variants that can be candidates for drug-resistance prediction.

### Calculation of sensitivity, specificity, and predictive values

We assessed the accuracy of our resistance prediction compared to standard pDST (ie, the overall probability that a sample was correctly classified as susceptible or resistant). We calculated sensitivity, specificity, and positive and negative predictive values. A customised script in R version 4.1.1 was employed in the calculations, following the classic mathematical formula for each parameter (appendix 1 p 2). The 95% CIs were calculated with the R package PropCIs (verson 0.3-0) using the function exact CI which calculates the Clopper–Pearson exact CIs.

### Role of the funding source

The funders of the study had no role in study design, data collection, data analysis, data interpretation, or writing of the report.

## Results

We performed WGS on 785 *M tuberculosis* culture-positive isolates from tuberculosis cases that were reported between Jan 1, 2014, and Dec 31, 2016, achieving a mean coverage depth of 130×. Five samples with cross contamination with other species were discarded from further analyses. After excluding 74 samples without epidemiological information or phenotypic data, 706 routine *M tuberculosis* samples remained, each representing an individual patient (appendix 2).

Full first-line drug susceptibility profiles were determined for 693 of the isolates by the hospitals. Of those, 626 (90·3%) were susceptible to all first-line anti-tuberculosis agents. The 13 isolates that were partially characterised also did not present any resistance. 55 (7·9%) cases were monoresistant: 29 (4·2%) were monoresistant to isoniazid, four (0·6%) to rifampicin, eight (1·2%) to ethambutol, and 14 (2·0%) to pyrazinamide. 11 (1·6%) of the cases were resistant to both isoniazid and rifampicin and only one (0·1%) isolate was polyresistant to isoniazid, ethambutol, and pyrazinamide.

**Figure 1:**
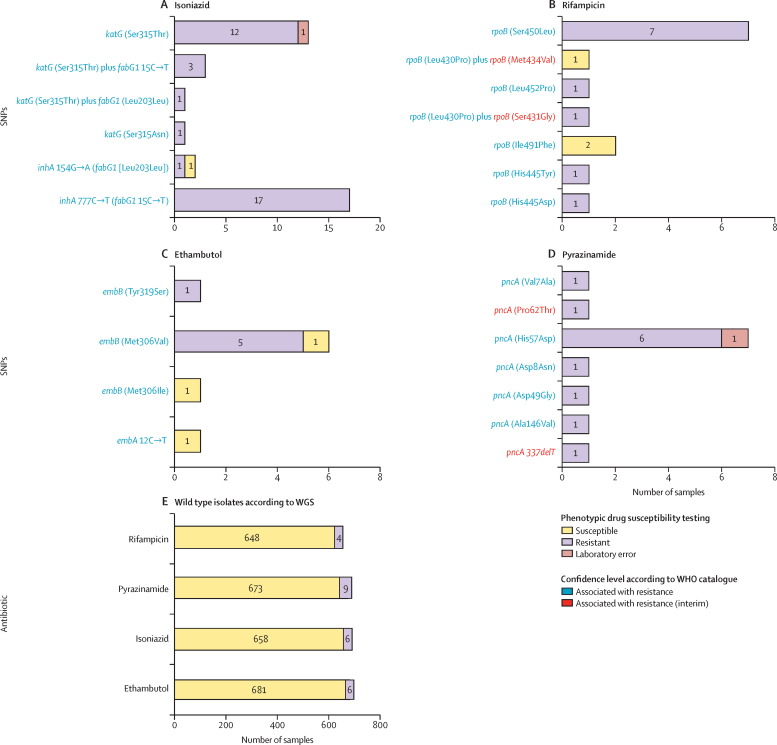
Frequency of resistance-conferring mutations for all first-line antituberculosis drugs Drug-resistant conferring variants identified by WGS in the dataset are classified depending on their association with resistance to isoniazid (A), rifampicin (B), ethambutol (C), and pyrazinamide (D). Variants strongly associated with drug resistance and variants supported by less evidence according to WHO are indicated. It is also indicated whether the mutation is present in a phenotypically resistant isolate or a susceptible isolate. SNP=single-nucleotide polymorphism. WGS=whole-genome sequencing.

According to our genome-based resistance prediction, 656 (92·9%) isolates were susceptible to the four first-line drugs. 39 (5·5%) cases were monoresistant, with 26 (3·7%) classified as monoresistant to isoniazid, three (0·4%) to rifampicin, three (0·4%) to ethambutol, and seven (1·0%) to pyrazinamide. Additionally, we found 11 (1·6%) of 706 isolates monoresistant to fluoroquinolones. There were 11 (1·6%) multidrug-resistant tuberculosis cases, and no isolates with polyresistance were detected. Two of the isolates had extremely well characterised resistance-associated mutations (*katG* Ser315Thr for isoniazid and *pncA* His57Asp for pyrazinamide) but were recorded as being susceptible by culture DST. We considered these pDST results very likely to be laboratory errors and excluded those isolates from further analyses. Three (0·45%) of 706 isolates were of lineage 1, 17 (1·7%) were of lineage 2, 19 (1·7%) were of lineage 3, 652 (94%) were of lineage 4, one (0·15%) was of lineage 5, and two (0·3%) were of lineage 6; lineage 7, lineage 8, and lineage 9 were not represented. Additionally, three patients were co-infected with two different sublineages of lineage 4. Among these samples we also identified some *M tuberculosis* complex pathogens different from *M tuberculosis*: seven (1·0%) of 706 were *Mycobacterium bovis* and two (0·3%) were *Mycobacterium caprae*. 62 (69·7%) of 89 phenotypic resistances in the dataset can be attributed to mutations strongly associated with resistance in the catalogue (figure 1). Discrepancies between genomic prediction and phenotype were found in 33 (4·7%) of 706 isolates. In some cases, different isolates with the same mutation showed different resistance phenotypes (figure 1). Only two samples presented mismatches for more than one antibiotic.

**Figure 2:**
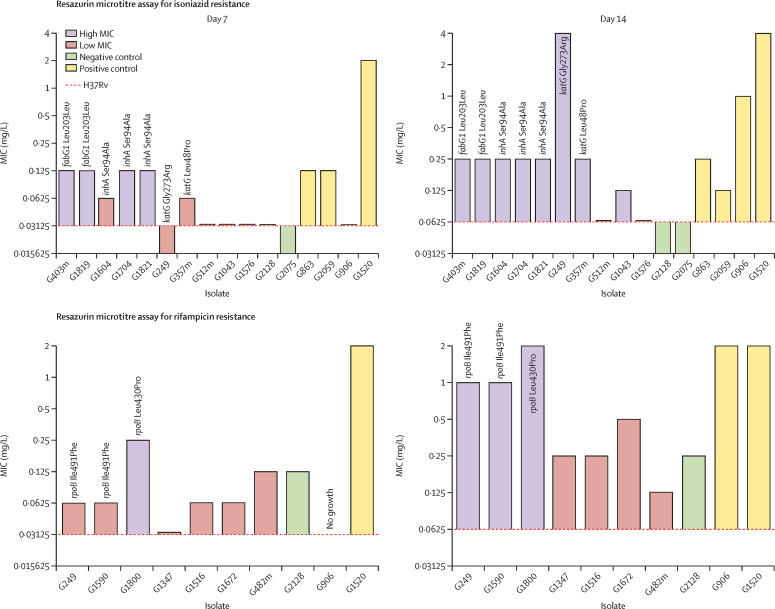
Variation of the isoniazid and rifampicin MIC of phenotype–genotype discrepant isolates in comparison to H37Rv The dashed horizontal line indicates the MIC of reference strain H37Rv. Non-discrepant isolates are also shown as a control. MICs are colour coded depending on whether they are more similar to those of the resistant control isolates or to those of the susceptible controls. Resistance-conferring mutations or most plausible resistance-associated mutations are indicated for each isolate, if present. MIC=minimum inhibitory concentration.

Only seven of the 71 predicted resistances were associated with a susceptible result in the pDST (appendix 1 p 4). Surprisingly, all seven isolates carried mutations related to resistance with a high degree of confidence. Additionally, two of these variants (*fabG1* Leu203Leu and *embB* Met306Val) were also present in phenotypically resistant isolates in the dataset. We determined the MICs for the antibiotic for which they had a resistance-conferring mutation of four of these seven isolates (isolate G1819 for isoniazid, and isolates G249, G1590, and G1800 for rifampicin) using the resazurin microtitre assay (figure 2; appendix 1 p 5). We measured the MIC at days 7 and 14 to account for the fact that resistance is sometimes associated with slow growth. All four strains showed a noticeable increase of the MIC in comparison with H37Rv and were closer to those of other resistant isolates in the dataset than to those of susceptible isolates, thus indicating that these isolates had some level of resistance and were at least borderline resistant. The variant *fabG1* Leu203Leu was found in G1819 and also in G403m, which had the same isoniazid MIC values (0·125 mg/L) but was resistant in pDST. For G249, G1590, and G1800, we detected the disputed *rpoB* mutations Leu430Pro and Ile491Phe. These isolates grow slowly and only after 14 days showed growth at a two-fold higher concentration than the current critical concentration for rifampicin (0·5 mg/L).

25 cases with phenotypic resistance were not associated with any mutation in the catalogue: six for isoniazid, four for rifampicin, six for ethambutol, and nine for pyrazinamide. Of these, 21 had no single-nucleotide polymorphisms or indels in known resistance genes (appendix 1 p 2) and all but one were monoresistant isolates. Three of the remaining isolates contained mutations that the WHO catalogue has classified as uncertain: *inhA* Ser94Ala (G1704) and *katG* Leu48Pro (G357) for isoniazid, and *pncA* Pro54Ala (G100) for pyrazinamide. *inhA* Ser94Ala was also present in two other isolates in this dataset that were susceptible to isoniazid (G1604 and G1821). Additionally, one isolate (G249) had a new putative mutation conferring resistance to isoniazid at 41% frequency: *katG* Gly273Arg.

We used the resazurin microtitre assay to determine the MICs of all the genotypically susceptible isolates with phenotypic resistance to either isoniazid or rifampicin, plus the two susceptible isolates with the *inhA* Ser94Ala mutation (figure 2). We found that the isolate with the *katG* Gly273Arg variant had a very high isoniazid MIC (0·015 mg/L at day 7 and 4 mg/L at day 14) but had a very low growth rate. Four isolates with the *inhA* Ser94Ala or *katG* Leu48Pro variants had MICs that were higher than those of susceptible isolates but lower than those of resistant isolates (figure 2), which means that they could be borderline resistance-associated mutations. All three isolates with *inhA* Ser94Ala had similar MICs, despite having different pDST results. However, it must be noted that all three are part of a transmission cluster. Finally, isolates with no candidate resistance-causing mutations showed very similar MICs to those of susceptible isolates (figure 2).

We explicitly evaluated the reliability of the WHO catalogue in predicting antimicrobial resistance profiles (table). Overall accuracy was very high, with over 98·7% of isolates showing concordant genotypic–phenotypic results. Sensitivity for detection of resistant cases ranged from 50·0% (95% CI 21·1–78·9) to 85·4% (70·8–94·4), showing that a sizable proportion of resistant isolates had no known resistance-associated mutations. Positive predictive value was high for isoniazid (97·2% [83·1–99·6]) and pyrazinamide (100·0% [73·5–100·0]) but modest for rifampicin (78·6% [53·2–92·2]) and ethambutol (66·7% [36·1–87·6]), implying that diagnostic mutations for those two antibiotics might not always be associated with a positive pDST. However, the catalogue can predict susceptibility with great accuracy: specificity values showed that over 99·5% of susceptible isolates were correctly detected and negative predictive values were 98·7% or above, which means that less than 1·5% of cases predicted to be susceptible to a given drug were actually resistant. Our ability to predict pan-susceptibility for the first-line antibiotics was very high, with an overall accuracy of 96·4%.

**Table:** Predictive values of whole-genome sequencing antibiotic resistance prediction with respect to standard pDST

	**Resistant cases according to pDST**	**Sensitivity**	**Specificity**	**Negative predictive value**	**Positive predictive value**	**Accuracy**
Isoniazid	41	85·4% (70·8–94·4)	99·8% (99·2–100·0)	99·1% (98·1–99·6)	97·2% (83·1–99·6)	99·0% (97·9–99·6)
Rifampicin	15	73·3% (44·9–92·2)	99·6% (98·6–99·9)	99·4% (98·6–99·7)	78·6% (53·2–92·2)	98·9% (97·8–99·6)
Ethambutol	12	50·0% (21·1–78·9)	99·6% (98·7–99·9)	99·1% (98·5–99·5)	66·7% (36·1–87·6)	98·7% (97·5–99·4)
Pyrazinamide	21	57·1% (34·0–78·2)	100·0% (99·4–100·0)	98·7% (97·9–99·2)	100·0% (73·5–100·0)	98·7% (97·5–99·4)

Data are n or % (95% CI). pDST=phenotypic drug susceptibility testing.

The latest WHO guidelines state that performing DNA sequencing to detect *rpoB* variants is preferred over culture-based DST, particularly in areas where it is likely to find mutations outside the rifampicin-resistance-determining region.^12^ We used this criterion to re-evaluate the resistance profile of some cases and we detected three new rifampicin-resistant cases. One of the new rifampicin-resistant cases contained the *rpoB* Leu430Pro mutation and the other two the *rpoB* Ile491Phe mutation. These single-nucleotide polymorphisms are both graded as associated with resistance by the WHO catalogue and are related to inconsistent results in pDST. As a result, two of the three isolates would now be considered multidrug-resistant tuberculosis and these patients should receive second-line treatment. Additionally, the WHO composite reference standard establishes that if an isolate is resistant to rifampicin and it harbours a mutation in *pncA*, it is also resistant to pyrazinamide, even though the variant is not included in the catalogue or has a low degree of confidence. In this case, the sample with Pro54Ala in *pncA* (classified as uncertain in the WHO catalogue) would be considered resistant to pyrazinamide.

## Discussion

In this population-based genomic study, we have shown that WGS resistance prediction using the first WHO catalogue substantially improves the detection of drug-resistant tuberculosis and allows for a better tailored treatment in a low-burden setting. Sequence-based prediction performs better than pDST for borderline resistance-associated mutations or variants associated with inconsistent phenotypes, thus increasing the ability to find resistant cases. Furthermore, genomic resistance analysis can predict resistance to second-line antibiotics not tested in standard pDST, which, as we have seen with our dataset, can be relevant even in low multidrug-resistant tuberculosis burden settings. Finally, routine use of WGS in clinical settings has the added benefit of incorporating new evidence for candidate resistance-conferring mutations or finding new ones.

The extremely high specificity of resistance prediction for all the first-line antibiotics shows the potential of WGS to rule out drug-resistant tuberculosis. However, low sensitivity is a striking finding in our study compared with other studies that have applied different customised mutation catalogues in similar clinical settings.^4^, ^14^, ^22^ We hypothesised that this finding might be a combined effect of having a low number of resistant isolates, and common resistance-conferring mutations not being over-represented either in the dataset or the population. However, lower sensitivities are not necessarily a feature of low-burden drug-resistant tuberculosis settings.^4^, ^14^ Apart from that, our resazurin microtitre assay results combined with the fact that we found no mutations in any of the relevant genes related to resistance suggest that in some cases the pDST result might be incorrect. It is important to consider that, despite being the gold standard, pDST is not error-free even when performed correctly and composite standards are becoming more recommended. This issue is further reinforced by the fact that most of these are monoresistant isolates for pyrazinamide and ethambutol.^23^ Errors that wrongly identify susceptible cases as resistant might lead to the unnecessary prescription of a second-line treatment.^24^ WGS could be used as a confirmatory tool in this circumstance, and if no plausible resistance-associated mutation is found the pDST should be repeated.

We identified two infrequent resistance-associated mutations in *katG* (Gly273Arg and Leu48Pro) and one in *inhA* (Ser94Ala) in three isoniazid monoresistant isolates initially predicted to be susceptible. One of them was not included in the catalogue (*katG* Gly273Arg), although there is strong evidence of its association with resistance.^25^ The other two variants were included in the catalogue as uncertainly related to resistance, even when there is evidence of *inhA* Ser94Ala providing resistance.^26^ Rare variants are frequently associated with specific lineages and restricted to geographical areas, or arise very rarely. For this reason, they are either poorly represented or even not included in most diagnostic mutation catalogues. This scarcity of global *M tuberculosis* diversity representation can mislead the performance of WGS genotyping, especially in high-burden settings and settings with high lineage diversity, whereby new and rare resistance mutations are more likely to be undetected.^27^, ^28^ This issue is why a reliable and up-to-date catalogue with special attention to region-specific particularities is extremely important in the development of genomic resistance prediction. Continued expansion and improvement of the catalogue via routine sequencing of isolates plus simplified reporting protocols would help us develop increasingly accurate diagnostics.

The WHO catalogue has been particularly useful for correctly predicting resistance for isolates with high-confidence resistance mutations that do not always show up as resistant in pDST. This phenomenon is not limited to our study but has been observed previously for mutations such as *fabG1* Leu203Leu, *rpoB* Leu430Pro, *embB* Met306Val, and *embB* Met306Ile.^14^, ^29^ When re-tested, isolates carrying those variants showed a borderline MIC and often slower replication. Thus, the most plausible explanation for inconsistent phenotypes is that some resistance-associated mutations confer borderline resistance to bacteria, according to the current critical concentrations or entail a fitness cost that diminishes the bacterial growth rate. These variants often lead to the incorrect identification of a resistant case as drug-susceptible and probably prescribing a less effective drug combination.^10^, ^30^

Even in a low-burden setting, we have identified relevant actionable clinical results. First, we have detected two unsuspected multidrug-resistant tuberculosis cases, which is invaluable information that can be used to give patients a better-tailored treatment.^25^ A second actionable result is the detection of resistance to antibiotics not tested in standard pDST. This finding is important because resistance to second-line antibiotics might arise due to incorrect treatment and for many second-line antibiotics testing is difficult and unreliable. In our results we have found an unexpectedly high frequency of fluoroquinolone monoresistant isolates, probably due to an over-prescription of fluoroquinolones. This finding illustrates how necessary a proper antibiotic stewardship is to reduce the emergence of resistances and how WGS can help identify such cases.

Our results are limited by the population structure of the bacteria in the region, dominated by lineage 4. Evidence is arising that lineage 1, which is uncommon in our setting, is associated with a higher basal MIC to pyrazinamide.^31^ The composition of lineages in different parts of the world is different and, although the catalogue was built from global collections, we cannot discard the effect of the bacterial diversity on sensitivity values. Moreover, in low-burden countries most cases of multidrug-resistant tuberculosis are imported, thus the diversity of drug-resistant strains and mutations will probably depend on the migrant population and their country of origin because drug-resistant strains have genetic differences in different parts of the world. However, in the Valencia Region, in Spain in general, and most countries with less than 10 in 100 000 cases the percentage of multidrug-resistant tuberculosis is very similar (mean of 1·77% between 2014 and 2016) with an overwhelming presence of pan-susceptible strains. Thus our results can probably be extrapolated to other low-burden regions, adding to the evidence already generated by other countries such as the UK^32^ and the Netherlands.^14^

In sum, our findings have value at different population levels. At the local level, our findings show that genome-based resistance prediction for the first-line antibiotics can complement pDST in routine diagnostics as the predictive power of the catalogue was overall excellent in our setting. In fact, WGS resistance prediction has the potential to enhance the tailoring of drug-resistant tuberculosis treatment as shown by the two unnoticed multidrug-resistant tuberculosis cases or the fluoroquinolone monoresistance cases. The local evidence generated in our study together with future updates of the catalogue will probably lead to the reduction of the use of culture as the front-line drug susceptibility method in our setting as has happened in other low-burden resistance settings.^4^, ^14^ At the global level, our findings show that the versatility of DNA sequencing also enables us to detect clerical errors, predict resistances to second-line antibiotics not included in routine pDST, and perform surveillance of antibiotic resistance.

## Data sharing

All information included in this study is provided in appendix 1 and appendix 2. Raw sequencing data is publicly available on the European Nucleotide Archive (https://www.ebi.ac.uk/ena/browser/home) using the accession numbers: PRJEB29604, PRJEB38719, PRJEB22237, and PRJEB70424. The accession numbers for each sample are included in appendix 2.

## Declaration of interests

IC received consultancy fees from the Foundation for Innovative New Diagnostics. IC and VF have participated in the elaboration of the WHO catalogue used in this study. All other authors declare no competing interests.
