# Supplementary material for: Role of the first WHO mutation catalogue in the diagnosis of antibiotic resistance in Mycobacterium tuberculosis in the Valencia Region, Spain: a retrospective genomic analysis
Source: Lancet Microbe. 2024 Jan;5(1):e43–51. doi: 10.1016/S2666-5247(23)00252-5 (PMC10790317; doi:10.1016/S2666-5247(23)00252-5)
Supplement: Supplementary appendix 1 [file mmc1.pdf]

THE LANCET
Microbe

Supplementary appendix 1

This appendix formed part of the original submission and has been peer reviewed. We post it as supplied by the authors.

Supplement to: García-Marín AM, Cancino-Muñoz I, Torres-Puente M, et al. Role of the first WHO mutation catalogue in the diagnosis of antibiotic resistance in *Mycobacterium tuberculosis* in the Valencia Region, Spain: a retrospective genomic analysis. *Lancet Microbe* 2023; published online Dec 4. https://doi.org/10.1016/S2666-5247(23)00252-5.

Supplementary Appendix 1

Content

Supplementary Methods

Sample collection and study design ……………………………………………………………… 1

Phenotypic drug susceptibility testing (pDST) …………………………………………………1

WGS and drug-susceptibility prediction ………………………………………………………..1-2

References …………………………………………………………………………………………2

Supplementary Figures

Figure S1. Flowchart of MTBC sample selection process ……………………………………….. 3

Supplementary Tables

Table S1. Clinical samples with phenotype-genotype discordances in resistance for Isoniazid, Rifampicin, Ethambutol or Pyrazinamide …………………………………………………………………. 4

Table S2. Isoniazid and rifampicin Minimum Inhibitory Concentration of the samples with phenotype-genotype discrepancies in resistance ……………………………………………………………5

Supplementary Methods

Sample collection and study design

All the Mycobacterium tuberculosis complex (MTBC) isolates were collected in clinical laboratories belonging to regional public hospitals. Twenty-five out of twenty-nine public hospitals in the Valencia Region are part of the local population-based genomic study. Aiming to collect as many culture-positive samples as we could, we ensured that all the major hospitals were included in the study since most hospitals refer the suspected TB samples to them.

All the cases were reported by regional public health authorities between Jan 1, 2014 and Dec 31, 2016, although the date of the culture positive sample may precede or postcede the period. Some samples may have a date from 2013 or 2017 because notification of the case to Public Health occurred during the study period but samples were taken or final result given before/after the period. The exact dates of the index tuberculosis cases are publicly available by Cancino-Muñoz et al.[1]

For isolates there was no pre-specified inclusion/exclusion criteria apart from being associated with a notified TB case in the region over the study period. We enrolled all notified TB cases from participating hospitals with available culture positive according to the standard practice in the hospital (MTBC cultures are tested by liquid Mycobacteria Growth Indicator Tube (MGIT) or solid-media Löwenstein-Jensen). For the downstream analysis we had to exclude some isolates because i. we did not confirm the presence of Mtb or they were contaminated (thus no genome sequence data could be recovered) or ii. they did not have associated susceptibility data to compare genotype-phenotype. We sequenced 785 single-patient samples, comprising 77·0% (785/1019) of all isolates that were reported culture-positive and 60·7% (785/1292) of all active TB cases notified in the region (Figure S1, appendix 1 p 3). After discarding all clinical samples that lacked pDST data from the laboratories and all sequence not belonging to MTBC complex, 706/785 (89·9%) isolates were included for further analysis.

Phenotypic drug susceptibility testing (pDST)

Phenotypic DST for first-line antibiotics was performed by the peripheral BSL3 clinical laboratories for routine patient-care purposes using standard procedures. Depending on the laboratory, we used either the proportion method on Löwenstein-Jensen media or the automated BACTEC MGIT 960 system (Becton Dickinson and Co, Franklin Lakes, New Jersey) with the manufacturer recommended critical concentrations for each drug: RIF (1·0μg/ml), INH (0·1μg/ml), ethambutol (EMB) (5·0μg/ml), pyrazinamide (PZA) (100μg/ml). In some cases, the phenotypic result was complemented using the molecular tests Genotype (HAIN Lifescience) and Anyplex™ MTB/MDR/XDR (Seegene).

WGS and drug-susceptibility prediction

The pipeline employed for the bioinformatic analysis works as follows. MTBC reads were mapped and aligned to an inferred MTBC most likely common ancestor genome using BWA.[2] SNPs with at least 10 reads in both strands and a quality score of 20 were selected and divided into: fixed SNPs (frequency of 90% or more) and low-frequency SNPs (frequencies range between 10% and 89%). An INDEL was considered when the mutation was present with a minimum depth coverage of 10x. SNP annotation was performed using H37Rv annotation reference (GenBank accession: AL123456.2). The automated screening includes positions in *katG*, *inhA* and its promoter, *fabG1,* and the *ahpC* promoter for isoniazid; *rpoB*, *rpoC* and *rpoA* for rifampicin; *embC*, *embA* and its promoter, and *embB* for ethambutol; *pncA* and its promoter, and *rpsA* for pyrazinamide.

Once the predictive variants were detected, we determined the resistance profile of each isolate. In order to establish the susceptible/resistant status for each antibiotic, the predictive variants were classified according to the grading of the WHO catalogue: associated with drug resistance, interim associated with drug resistance, uncertain significance, not associated with drug resistance, and not associated with drug resistance interim.[3] Those isolates with mutations graded as associated with drug resistance or interim associated with drug resistance were predicted genotypically resistant and those ones wich lacked any of these mutations or with 'uncertain significance' mutations were reported as susceptible. Additionally, we followed the expert rules stated in the catalogue, which i. change the status of some "Uncertain significance" mutations to "Associated" or ''Associated - Interim" based on expert knowledge and ii. consider any isolate carrying a nonsense mutation or indel in the coding region of specific genes as resistant, even if the variant is not listed in the catalogue.

As the association with resistance of the variants *katG* S315T and *pncA* H57D has been extensively demonstrated, isolates with these SNPs but reported phenotypically drug-susceptible were excluded from further analyses. The variant *katG* S315T confers relatively large MIC increases and should, therefore, test reliably resistant. [4] The *pncA* H57D variant has been described for causing intrinsic pyrazinamide resistance which is representative of *Mycobacterium bovis*. Thus, isolates identified as *M. bovis* carrying this mutation should test resistant to pyrazinamide. [5] This is why these discrepancies are assumed to be very likely laboratory errors and this is usually stated as an exclusion criteria in sensitivity and specificity determinations.[6] Additionally, we considered isolates with three or more phenotype-genotype discrepancies as clerical errors.

Additionally, in those isolates with phenotype-genotype discrepancies, we scanned the genes from the resistance catalogue and a secondary list of genes strongly associated with resistance according to it [3]: *ndh*, *kasA*, Rv1258c, and Rv2752c for isoniazid resistance; *rpoZ,* and Rv2752c for rifampicin resistance; *embR*, *ubiA*, *manB*, *iniC*, *iniA,* and *rmlD* for ethambutol resistance; *clpC1*, *panD*, PPE35, Rv1258c, and Rv3236c for pyrazinamide resistance.

Calculation of sensitivity, specificity and predictive values

Sensitivity, specificity, and positive and negative predictive values were calculated using the classical formulas applied with a customized R script. The 95% confidence intervals were calculated using a package in R called PropCIs. This package includes the function exactCI which calculates the Clopper-Pearson exact CIs. These are all the formulas included in the script:

- True Positives: isolates which are resistant according pDST and WGS prediction
- True Negatives: isolates which are susceptible according pDST and WGS prediction
- False Positives: isolates which are resistant only according WGS prediction
- False Negatives: isolates which are susceptible only according WGS prediction

$$\text{Sensitivity} = TP / (TP + FN)$$

$$\text{Specificity} = TN / (TN + FP)$$

$$\text{Positive Predictive Value} = TP / (TP + FP)$$

$$\text{Negative Predictive Value} = TN / (TN + FN)$$

References

1 Cancino-Muñoz I, López MG, Torres-Puente M, *et al.* Population-based sequencing of Mycobacterium tuberculosis reveals how current population dynamics are shaped by past epidemics. eLife 2022; **11**:e76605

2 Li H, Durbin R. Fast and accurate short read alignment with Burrows-Wheeler transform. *Bioinformatics* 2009; **25**: 1754–60.

3 WHO. Catalogue of mutations in Mycobacterium tuberculosis complex and their association with drug resistance. Geneva: World Health Organization; 2021 [cited 2021 Sep 24]. Available from: https://www.who.int/publications/i/item/9789240028173

4 Ghodousi A, Tagliani E, Karunaratne E, *et al.* Isoniazid Resistance in Is a Heterogeneous Phenotype Composed of Overlapping MIC Distributions with Different Underlying Resistance Mechanisms. *Antimicrob Agents Chemother* 2019; **63**:e00092-19.

5 Scorpio A, Zhang Y. Mutations in pncA, a gene encoding pyrazinamidase/nicotinamidase, cause resistance to the antituberculous drug pyrazinamide in tubercle bacillus. *Nat Med* 1996; **2**: 662–7.

6 CRyPTIC Consortium and the 100,000 Genomes Project, Allix-Béguec C, Arandjelovic I, *et al.* Prediction of Susceptibility to First-Line Tuberculosis Drugs by DNA Sequencing. *N Engl J Med* 2018; **379**: 1403–15.

Figure S1. Flowchart of MTBC sample selection process

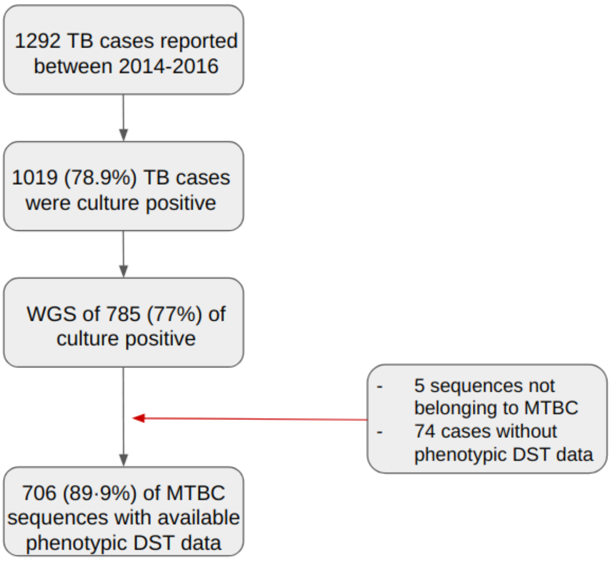

Table S1. Clinical samples with phenotype-genotype discordances in resistance for Isoniazid, Rifampicin, Ethambutol or Pyrazinamide.

Sample ID	Type	Antibiotic	pDST original	Resistance-conferring mutation	Grade in WHO catalogue 2021
G249	FN	Isoniazid	Resistant	*katG G273R*	Not in catalogue
G357m	FN	Isoniazid	Resistant	*katG L48P*	Uncertain significance
G1043	FN	Isoniazid	Resistant	None	-
G512m	FN	Isoniazid	Resistant	None	-
G1576	FN	Isoniazid	Resistant	None	-
G1704	FN	Isoniazid	Resistant	*inhA S94A*	Uncertain significance
G1819	FP	Isoniazid	Susceptible	*inhA g-154a (fabG1_L203L)*	Associated with resistance
G1347	FN	Rifampicin	Resistant	None	-
G1516	FN	Rifampicin	Resistant	None	-
G1672	FN	Rifampicin	Resistant	None	-
G428m	FN	Rifampicin	Resistant	None	-
G249	FP	Rifampicin	Susceptible	*rpoB* I491F	Associated with resistance
G1590	FP	Rifampicin	Susceptible	*rpoB* I491F	Associated with resistance
G1800	FP	Rifampicin	Susceptible	*rpoB L430P*	Associated with resistance
G102	FP	Ethambutol	Susceptible	*embA* c-12t	Associated with resistance
G486	FP	Ethambutol	Susceptible	*embB* M306I	Associated with resistance
G862m	FP	Ethambutol	Susceptible	*embB* M306V	Associated with resistance
G1487	FN	Ethambutol	Resistant	None	-
G1526	FN	Ethambutol	Resistant	None	-
G1538	FN	Ethambutol	Resistant	None	-
G1654	FN	Ethambutol	Resistant	None	-
G287	FN	Ethambutol	Resistant	None	-
G757m	FN	Ethambutol	Resistant	None	-
G100	FN	Pyrazinamide	Resistant	*pncA* P54A	Uncertain significance
G1280	FN	Pyrazinamide	Resistant	None	-
G145	FN	Pyrazinamide	Resistant	None	-
G1702	FN	Pyrazinamide	Resistant	None	-
G1900	FN	Pyrazinamide	Resistant	Rv3236c_A370T	Not associated with resistance
G222	FN	Pyrazinamide	Resistant	None	-
G356m	FN	Pyrazinamide	Resistant	None	-
G562m	FN	Pyrazinamide	Resistant	None	-
G788FE29	FN	Pyrazinamide	Resistant	None	-

TP = True Positive; TN = True Negative; FP = False Positive; FN = False Negative; pDST = phenotypic Drug Susceptibility Testing;

Table S2. Isoniazid and rifampicin Minimum Inhibitory Concentration of the samples with phenotype-genotype discrepancies in resistance.

Sample	Type	Antibiotic	pDST original	MIC Day 8 (µg/mL)	MIC day 15 (µg/mL)	Critical concentration (µg/mL)	REMA result	Resistance-conferring mutation	Grade in WHO catalogue 2021
G249	FN	Isoniazid	Resistant	0·0156	4	0·1	Resistant	*katG* G273R	Not in catalogue
G357m	FN	Isoniazid	Resistant	0·0625	0·25	0·1	Resistant	*katG* L48P	Uncertain significance
G403m	TP	Isoniazid	Resistant	0·1250	0·25	0·1	Resistant	*inhA g-154a (fabG1_L203L)*	Associated with resistance
G1043	FN	Isoniazid	Resistant	0·0312	0·125	0·1	Susceptible	None	-
G512m	FN	Isoniazid	Resistant	0·0312	0·0625	0·1	Susceptible	None	-
G1576	FN	Isoniazid	Resistant	0·0312	0·0625	0·1	Susceptible	None	-
G1704	FN	Isoniazid	Resistant	0·1250	0·25	0·1	Resistant	*inhA* S94A	Uncertain significance
G1604	TN	Isoniazid	Susceptible	0·0625	0·25	0·1	Resistant	*inhA* S94A	Uncertain significance
G1821	TN	Isoniazid	Susceptible	0·1250	0·25	0·1	Resistant	*inhA* S94A	Uncertain significance
G1819	FP	Isoniazid	Susceptible	0·1250	0·25	0·1	Resistant	*inhA g-154a (fabG1_L203L)*	Associated with resistance
G212	TN	Isoniazid	Susceptible	0·0156	0·0625	0·1	Susceptible	None	-
H37Rv	Control	Isoniazid	Quality Control	0·0312	0·0625	0·1	Susceptible	None	-
H37Rv	Control	Isoniazid	Quality Control	0·0312	0·0625	0·1	Susceptible	None	-
G2128	TN	Isoniazid	Susceptible	0·0312	0·0312	0·1	Susceptible	None	-
G2075	TN	Isoniazid	Susceptible	0·0156	0·03125	0·1	Susceptible	None	-
G863	TP	Isoniazid	Resistant	0·1250	0·25	0·1	Resistant	*inhA* c-777t (*fabG1* c-15t)	Associated with resistance
G2059	TP	Isoniazid	Resistant	0·1250	0·125	0·1	Resistant	*inhA* c-777t (*fabG1* c-15t)	Associated with resistance
G906	TP	Isoniazid	Resistant	0·0000	1	0·1	Resistant	*katG* S315T	Associated with resistance
G1520	TP	Isoniazid	Resistant	2·0000	4	0·1	Resistant	*katG* S315T	Associated with resistance
G1347	FN	Rifampicin	Resistant	0·1250	0·25	0·5	Susceptible	None	-
G1516	FN	Rifampicin	Resistant	0·1250	0·25	0·5	Susceptible	None	-
G1672	FN	Rifampicin	Resistant	0·1250	0·25	0·5	Susceptible	None	-
G428m	FN	Rifampicin	Resistant	0·1250	0·25	0·5	Susceptible	None	-
G249	FP	Rifampicin	Susceptible	0·2500	1	0·5	Resistant	*rpoB* I491F	Associated with resistance
G1590	FP	Rifampicin	Susceptible	0·5000	2	0·5	Resistant	*rpoB* I491F	Associated with resistance
G1800	FP	Rifampicin	Susceptible	1·0000	2	0·5	Resistant	*rpoB* L430P	Associated with resistance
H37Rv	Control	Rifampicin	Quality Control	0·0312	0·0625	0·5	Susceptible	None	-
H37Rv	Control	Rifampicin	Quality Control	0·0312	0·0625	0·5	Susceptible	None	-
G2128	TN	Rifampicin	Susceptible	0·1250	0·25	0·5	Susceptible	None	-
G2075	TN	Rifampicin	Susceptible	0·0156	0·03125	0·5	Susceptible	None	-
G143	TP	Rifampicin	Resistant	0·5000	2	0·5	Resistant	*rpoB* L452P	Associated with resistance
G906	TP	Rifampicin	Resistant	2	2	0·5	Resistant	*rpoB* H445Y	Associated with resistance
G1520	TP	Rifampicin	Resistant	2	2	0·5	Resistant	*rpoB* S431G	Associated with resistance Interim

TP = True Positive; TN = True Negative; FP = False Positive; FN = False Negative; pDST = phenotypic Drug Susceptibility Testing; MIC = Minimum Inhibitory Concentration
